# 516 Quantitation of Contact Pathway Activators Before and After Fresh Frozen Plasma Administration in Burn Patients

**DOI:** 10.1093/jbcr/iraf019.145

**Published:** 2025-04-01

**Authors:** Samuel DiPasquale, Maria Cristina Bravo, Meghan Fondakowski, Matthew Gissel, Lauren Moffatt, Melissa McLawhorn, Anthony Pusateri, Jeffrey Shupp, Thomas Orfeo

**Affiliations:** University of Vermont; University of Vermont; University of Vermont; University of Vermont; MedStar Health Research Institute; MedStar Health Research Institute; Naval Medical Research Unit San Antonio; MedStar Washington Hospital Center; University of Vermont

## Abstract

**Introduction:**

Major thermal injury induces profound systemic responses including release of cellular debris into the blood. The potential of certain types of cellular debris to support activation of the contact pathway via promoting auto-activation of FXII is well established. However, studies quantifying the levels of such “activating surfaces” in plasma from burn patients are lacking. Here we report functional levels of such material in burn patients before and after the administration of fresh frozen plasma (FFP).

**Methods:**

Patients (n=23) were prospectively enrolled in an IRB approved study. Platelet poor plasma was prepared from blood samples collected from patients (TBSA 40±19%, range 19-81%) prior to administration of the first transfused FFP unit (BD1: time from injury 383 ± 249 min, range 187-1153 min) and after transfusion of 1 FFP unit (BD 2: 25 ± 11 min post BD1; range 11-57 min). Plasma samples from patients and administered FFP units were thawed in the presence of corn trypsin inhibitor (to block FXII activation during thaw) and large particulates separated from other plasma components via gel filtration. Contact pathway activation potential in gel filtration isolates was quantified by combining purified zymogens (fXII, prekallikrein, high molecular weight kininogen (HMWK) under assay conditions where FXIIa formation and kallikrein formation are only observed when both HMWK and an activating surface are present in the reaction. Kallikrein formation is monitored via chromogenic substrate and rates of kallikrein formation by isolates were translated into relative units via a standard curve constructed using a commercial contact pathway activator (Intem) assigned a relative concentration of 1 U/mL. Data are presented as mean ± SD; paired t-tests were used to compare findings across sampling times and unpaired between patient samples and FFP units.

**Results:**

The concentrations of contact pathway activators at BD1 (9.0 ± 6.5 mU/mL, range 2-24) did not change in response to FFP administration (8.4 ± 6.3 mU/mL, range 1-24). FFP units (n=20) showed a significantly lower concentration of contact pathway activator (3.7 ± 3.8 mU/mL, range 1-28, p≤0.005 vs both time points).

**Conclusions:**

Material capable of promoting contact activation is present at elevated levels in burn patient plasma relative to FFP from healthy donors. A single transfusion unit of FFP does not alter the level of contact pathway activators detected in burn patient plasma.

**Applicability of Research to Practice:**

Coagulopathy represents a common challenge to the management of severely burned patients. Understanding the degree to which contact pathway activation contributes to hemostatic derangements could inform therapeutic approaches.

**Funding for the Study:**

USAMRAAA - BA190086

